# Pattern of use and awareness of side-effects of non-steroidal anti-inflammatory drugs in the Jordanian population

**DOI:** 10.1080/07853890.2023.2242248

**Published:** 2023-08-01

**Authors:** Randa I. Farah, Aseil E. Khatib, Hiba J. Abu Ziyad, Dareen K. Jiad, Lara R. Al Qusous, Ali Jamal Ababneh, Salma Ajarmeh

**Affiliations:** a Nephrology Division, Department of Internal Medicine, School of Medicine, University of Jordan, Amman, Jordan; b School of Medicine, University of Jordan Hospital, Amman, Jordan; c School of Pharmacy, University of Jordan, Amman, Jordan; d Department of Pediatrics, School of Medicine, Mutah University, Karak, Jordan

**Keywords:** Anti-inflammatory agents, non-steroidal, drug utilization, drug-related side-effects and adverse reactions, drug interactions, pharmacovigilance, awareness

## Abstract

**Background:** Non-steroidal anti-inflammatory drugs (NSAIDs) are commonly utilized to reduce pain, inflammation, and fever. This study aimed to assess patterns of use and awareness of NSAID-related side-effects in an adult Jordanian. And the associations with sociodemographic factors. **Methods:** This cross-sectional study among a representative sample of 604 adults >18 years. A validated, self-administered questionnaire was used to collect basic sociodemographic data from the participants, as well as information regarding NSAID use. **Results:** Most respondents were NSAID users (65.7%), female (53.4%) and under 50 years of age (74.5%). Overall, 42.6% had been prescribed NSAIDs by a physician. Male gender and smoking were negatively correlated with NSAIDs use (multivariable odds ratio [OR]: 0.5, 95% confidence interval [CI]: 0.4–0.8, *p* = 0.001 and OR: 0.6, 95% CI 0.4–0.8, *p* = 0.003). In contrast, the Ministry of Health Insurance was associated with NSAIDs use with OR: 1.6, 95% CI: 1.1–2.6, *p* = 0.03. Overall, 65.1% were aware of kidney NSAID-related side-effects and 22.4% were aware of the increased risk of asthma and allergy. **Conclusion:** Despite the high frequency of NSAID use in the Jordanian general population, there is limited knowledge of their side-effects as well as drug interactions. This is cause for concern, particularly as many participants reported having been prescribed NSAIDs by physicians without adequate patient safety education.

## Introduction

Non-steroidal anti-inflammatory drugs (NSAIDs) are a group of medications that act as painkillers and reduce fever and inflammation but have concerning side-effects [1]. These medications are used to treat different conditions, including headache, fever, acute and chronic pain, biliary and ureteric colic, rheumatologic diseases, and dysmenorrhea. Such drugs are prescribed worldwide in clinics and hospitals and can also be purchased over the counter (i.e. without a prescription), as their use is believed to be safe even over long periods [2]. Moreover, purchasing an analgesic without a prescription is considered by many members of the public to be less time-consuming, less costly, and result in faster pain relief compared to visiting an emergency room or making an appointment to be examined by a general physician [3].

Nonetheless, it is vital that members of the public who use NSAIDs over a long period of time are aware of the maximum daily dose permissible and which side-effects can be harmful. Many adverse reactions can occur following NSAID use, including in the gastrointestinal (GI) tract, kidney, liver, skin, central nervous system, and blood [4]. According to a study conducted in an emergency department in California, most users (61%) are aware of the GI side-effects of NSAID use, while only a small proportion understand that utilization of these drugs can result in specific kidney problems and other adverse effects [5].

As such, the aim of this study was to assess the pattern of utilization of NSAIDs and level of awareness regarding NSAID-related side-effects among a representative sample of the Jordanian public. In addition, the study aimed to determine factors affecting NSAID use and awareness, such as sociodemographic factors, personal health, and family history of illness. It is important to assess the general public’s knowledge of NSAIDs and their related side-effects, as their misuse may negatively impact health. A study conducted among medical students in India demonstrated that while most students were familiar with over-the-counter-medications, very few demonstrated sufficient knowledge regarding the regulation and usage of self-medication [6].

## Methodology

### Study design, inclusion, and sample procedure

This is a cross-sectional study conducted in two major shopping centers in Amman, the capital of Jordan. Data was collected between June 2021 and September 2021. A questionnaire was distributed by medical students to the target population which included the public over 18 years of age. Healthcare providers were excluded. After an introduction about the purpose of the study and the nature of the questionnaire, the participants provided written consent before filling out the questionnaire. The questionnaire was self-administered, and non-influential assistance was provided by trained medical students, if requested. The sample size required to achieve a 95% CI was calculated with a margin of error of 5%. Assuming a response rate of 60%, we concluded that the sample should be 369 or more participants. A total of 604 Jordanian general public participants answered the questionnaire.

### Study questionnaire

A validated, self-administered questionnaire was used to collect basic sociodemographic data from the participants, as well as information regarding NSAID usage, patterns of use, and knowledge of their potential side-effects. The self-administered questionnaire was divided into three sections. The first section assessed the participants’ sociodemographic information, including their gender, age, marital status, level of education, type of work, insurance type, smoking status, and body mass index. The second part included relevant medical history, current medication use, and frequency of administration. The third and final part of the questionnaire sought details regarding the respondent’s use of NSAIDs. After providing participants with a list of all NSAIDs brands used in Jordan, we collected the following data: purpose of use, whether drugs were purchased without prescriptions or prescribed by a physician or pharmacist, and awareness of potential side-effects associated with NSAIDs, where a list of potential side effects was provided.

The questionnaire was validated using a pilot study of 20 participants and reviewed by two academically oriented physicians. This provided feedback regarding the cohesion and coherence of the instrument, as well as its clarity and ease of use. During the main study, investigators assisted respondents in answering the questionnaire to minimize the possibility of respondents not understanding specific questions.

### Statistical analysis

Data analysis was performed using the Statistical Package for the Social Sciences (SPSS) software, version 25 (IBM Corp., Armonk, New York, USA). Results were summarized using descriptive statistics, including frequencies (*n*) and percentages (%). A Chi-squared test (χ^2^) was used to assess the associated factors with NSAIDs use then the multivariable logistic regression model was constructed to identify variables associated with NSAIDs use. Only significant variables with P value < 0.05 was considered significant were included in the final multivariable regression model. A Chi-squared test (χ^2^) was used to determine the difference in the NSAIDs side effects awareness between NSAIDs users and non-user, with a *p* value of <0.05 considered to indicate statistically significant differences. During analysis, when assessing the relationship between NSAID use and awareness of its side-effects, respondents were divided into four categories: (1) individuals who were aware of side-effects and who continued to use these medications; (2) those who did not use these medications and knew of their side-effects; (3) those who neither used such medications nor knew of their side-effects; and (4) those who used these medications, but did not know of their side-effects.

## Results

A total of 604 members of the Jordanian general public were surveyed, 450 (74.5%) participants were younger than 50 years and 260 (43%) were smokers. In regards to comorbidities, 15.7% of participants have hypertension and 10.9% have diabetes. Also in this study, 22.8% of participants don’t have any medical insurance while 35.3% have Ministry of Health Insurance. The NSAIDs users were 397 (65.7%), the majority of them were females (56.7%) (p value = 0.03). In addition, 46.9% of the NSAID users’ group were smokers compared to 35.7% of non-NSAIDs users (p value = 0.03). Of the NSAIDs users,53 (13.4%) have hypertension compared to 20.3% of non-NSAIDs users (*p* = 0.03), and 8.8% of NSAIDs users have diabetes compared to 14.9% with a p value = 0.02 (Table 1).

**Table 1.** Factors associated with NSAIDs use (univariate associations).

Baseline characteristics		Total no. 604 (%)	NSAIDs users (%)	Non-NSAIDs user (%)	*p* Value
Gender	Female	323 (53.4)	225 (56.7)	98 (47.3)	0.029*
	Male	281 (46.5)	172 (43.3)	109 (52.7)	
Age	<50	450 (74.5)	303 (76.3)	147 (71.0)	0.155
	> =50	154 (25.5)	94 (23.7)	60 (29.0)	
Social status	Married	343 (56.8)	223 (56.2)	120 (57.9)	0.914
	Single	246 (40.7)	164 (41.3)	82 (39.6)	
	Other	15 (2.5)	10 (2.5)	5 (2.4)	
Education level	Middle school	47 (7.8)	26 (6.5)	21 (10.1)	0.100
	High school	162 (26.8)	98 (24.7)	64 (30.9)	
	Bachelor’s degree	354 (58.6)	245 (61.7)	109 (52.6)	
	Masters/PhD	41 (6.8)	28 (7.1)	13 (6.2)	
Working status	Student	131 (21.7)	81 (20.4)	50 (24.1)	0.066
	Working	292 (48.3)	204 (51.4)	88 (42.5)	
	Housewife	116 (19.2)	77 (19.4)	39 (18.8)	
	Retired	65 (10.7)	35 (8.8)	30 (14.4)	
Smoking status	Non-smoker	315 (52.1)	192 (49.4)	123 (59.4)	0.029*
	Smoker	260 (43)	186 (46.9)	74 (35.7)	
	Ex-smoker	29 (4.8)	19 (4.8)	10 (4.8)	
Insurance	Private	160 (26.5)	112 (28.2)	48 (23.2)	0.032*
	Ministry of Health	213 (35.3)	125 (31.5)	88 (52.5)	
	University	93 (15.4)	60 (15.1)	33 (15.9)	
	None	138 (22.8)	100 (25.2)	38 (18.3)	
Body-mass-index	Normal	208 (34.4)	142 (35.8)	66 (31.9)	0.809
	Underweight	52 (8.6)	33 (8.3)	19 (9.1)	
	Overweight	212 (35.0)	136 (34.3)	76 (36.7)	
	Obese	132 (21.8)	86 (21.7)	46 (22.2)	
Comorbidities	Hypertension	95 (15.7)	53 (13.4)	42 (20.3)	0.026*
	Diabetes mellitus	66 (10.9)	35 (8.8)	31 (14.9)	0.021*
	Gastrointestinal disease	31 (5.1)	23 (5.8)	8 (3.8)	0.308
	Heart disease	17 (2.8)	10 (2.5)	7 (3.3)	0.543
	Asthma	10 (1.7)	8 (2.0)	2 (1)	0.338
	Musculoskeletal disease	31 (5.1)	23 (5.8)	8 (3.8)	0.308
	Thyroid disease	28 (4.6)	16 (4.0)	12 (5.8)	0.327

NSAIDs: Non-steroidal anti-inflammatory drugs; *Significant value.

Table 2 shows the multivariable analysis evaluating factors associated with NSAIDs use. Male gender and smoking were negatively correlated with NSAIDs use (multivariable odds ratio [OR]: 0.5, 95% confidence interval [CI]: 0.4–0.8, *p* = 0.001 and OR: 0.6, 95% CI 0.4–0.8, *p* = 0.003). The Ministry of Health Insurance was associated with NSAIDs use with OR: 1.6, 95% CI: 1.1–2.6, *p* = 0.03 (Table 2).

**Table 2.** Multivariable logistic regression analyses of NSAIDs use.

Variable	Subgroups	Odd ratio	95% CI	*p* Value
Sex	Female (Ref)			
	Male	0.5	0.4–0.8	0.001*
Medical history	Diabetes	0.7	0.4–1.4	0.3
	Hypertension	0.9	0.5–1.5	0.6
Smoking	Non- smoker (Ref)			
	Smoker	0.6	0.4–0.8	0.003*
	Ex-Smoker	0.5	0.2–1.2	0.12
Medical Insurance	Private (Ref)			
	Ministry of Health	1.6	1.1–2.6	0.03*
	University insurance	1.4	0.8–2.5	0.2
	Non	0.9	0.6–1.6	0.8

CI: Confidence interval; *Statically significant.

With regards to awareness of NSAID-related side-effects, respondents were initially asked if they knew that NSAIDs could interact with each other or different medications, of which 37.9% affirmed that they could. As for organ- or system-specific side-effects, 65.1% and 52.6% of respondents were aware of possible kidney and GI problems, respectively, but only 22.4% were aware of the increased risk of asthma and allergy (Figure 2). Overall, only 29.6% of NSAID users reported having received patient education regarding the potential side-effects of these medications. In a univariate analysis comparing NSAIDs users with non-users, we found that non-NSAIDs users are more aware of the NSAID-related kidney and GI side-effects with a p value 0.001 and 0.004 respectively. We found no difference in awareness regarding NSAIDs related hypertension and heart disease side effects (*p* value = 0.3) (Figure 2 and Table 3).

**Table 3.** NSAIDs awareness questions (univariate associations).

Awareness questions	Total no.	NSAIDs users (%)	Non-NSAIDs user (%)	*p* Value
	604 (%)			
Do you know whether NSAIDs are safe in pregnancy? (Yes)	284 (47)	192 (48.4)	92 (51.6%)	0.4
Did anyone explain to you side effects of NSAIDs? (Yes)	179 (29.6)	129 (72.0)	50 (27.9)	0.03*
Are you aware NSAIDs may cause hypertension and heart disease? (Yes)	199 (32.9)	136 (68.3)	63 (31.7)	0.3
Are you aware NSAIDs may increase the risk of asthma or cause allergy? (Yes)	135 (22.4)	88 (65.2)	47 (34.8)	0.9
Are you aware NSAIDs may cause gastrointestinal problems? (Yes)	318 (52.6)	226 (71.1)	92 (28.9)	0.004*
Are you aware NSAIDs may cause kidney problems? (Yes)	393 (65.1)	279 (70.2)	117 (29.8)	0.001*
Are you aware NSAIDs can interact with each other and other drugs? (Yes)	229 (37.9)	142 (62)	87 (38)	0.1

*Statically significant.

**Figure 1.**
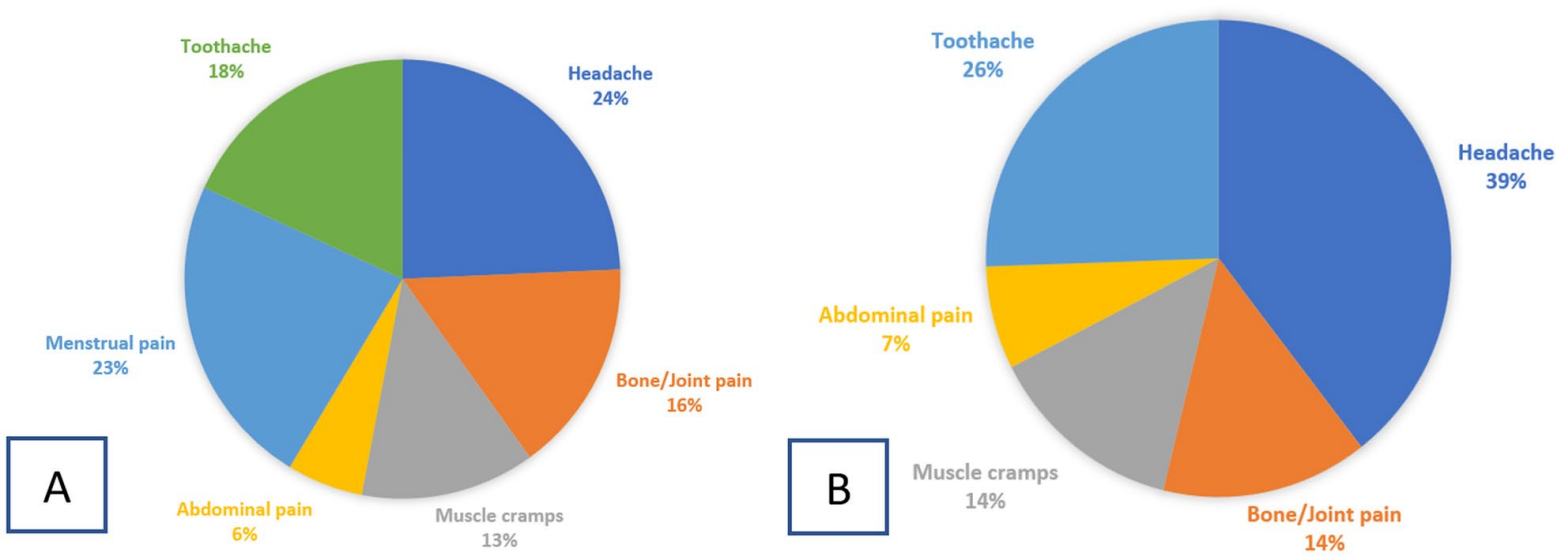
Indications for NSAID use among members of the Jordanian public. (A) Women. (B) Men. NSAID: non-steroidal anti-inflammatory drug.

Most users were prescribed NSAIDs by a physician (42.6%) or pharmacist (17%), while 28.6% were taking these medications without prescriptions. Only 29.6% of NSAID users were warned of the potential side-effects of these medications. Overall, headache was the most common indication for NSAID use (39% in men and 24% in women), followed by toothache (26%) in men and menstrual pain (23%) in women, while the least common indication was abdominal pain (7% in men and 6% in women) (Figure 1(A,B)).

**Figure 2.**
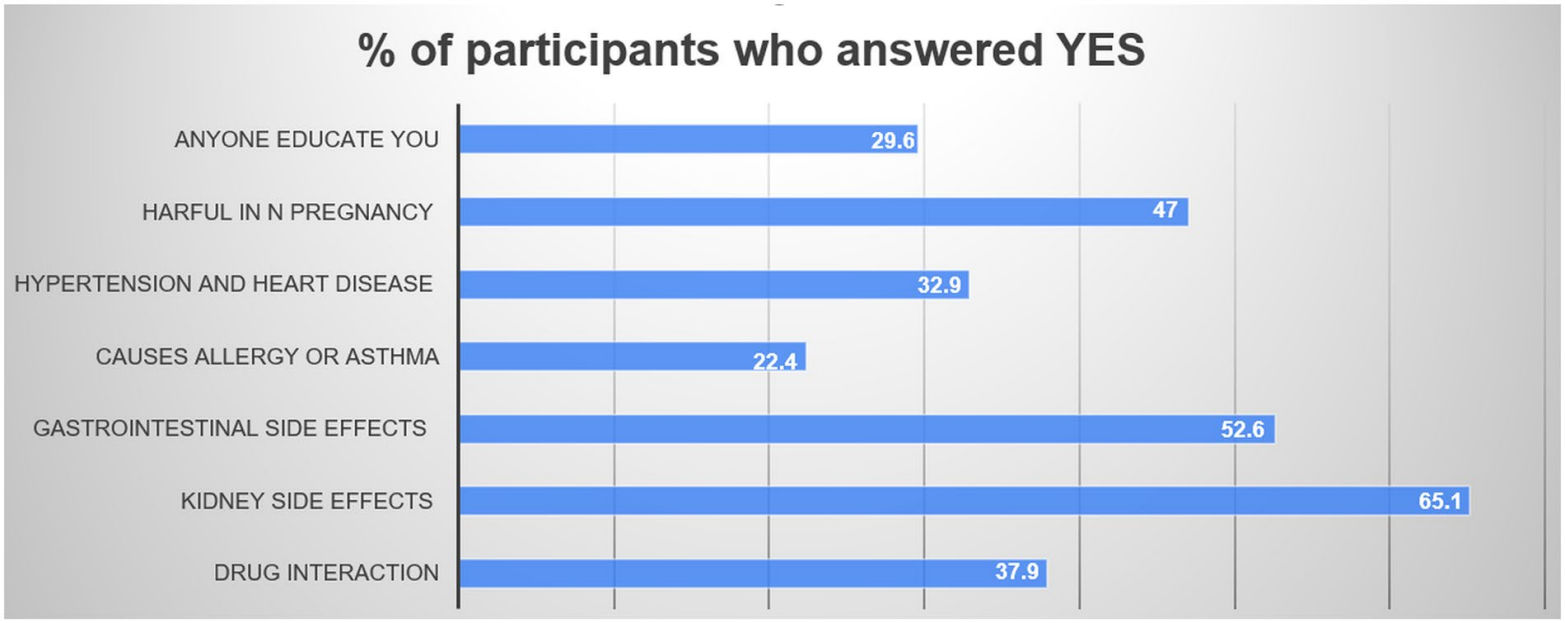
Awareness of NSAID-related side-effects among members of the Jordanian public. NSAID: non-steroidal anti-inflammatory drug.

Most respondents (81%) utilized NSAIDs once per month or less, with 10.5% using them once per week, 4.6% two to three times per week, and 3.7% on a daily basis. Factors associated with NSAID use included gender (*p* = 0.029) and type of health insurance (*p* = 0.032), with female respondents and those with government insurance more frequently utilizing NSAIDs compared to their respective counterparts. In turn, those who smoked or had a history of diabetes mellitus or hypertension were significantly less likely to use NSAIDs, with *p* values of 0.0.24, 0.026, and 0.021, respectively (Table 1).

**Figure 3.**
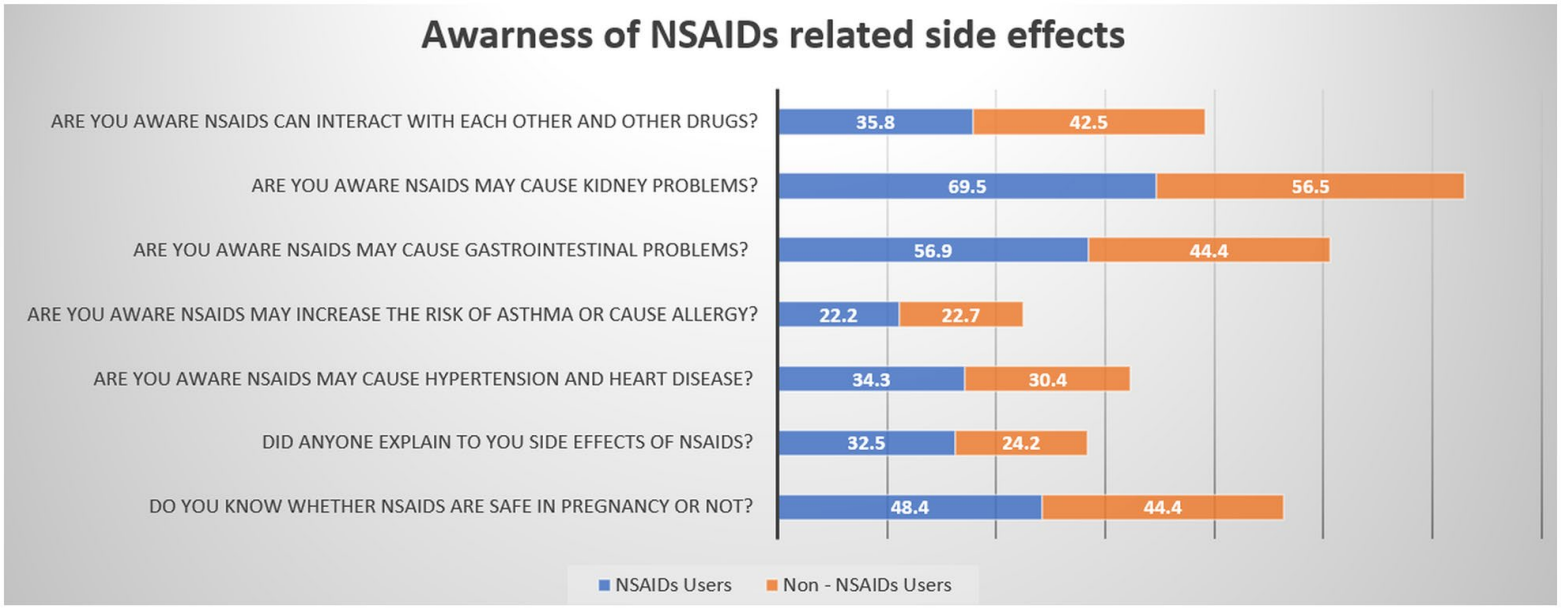
Awareness of NSAID-related side-effects among members of the Jordanian public according to patterns of utilization. NSAID: non-steroidal anti-inflammatory drug.

With regards to specific adverse effects, 51.2% of respondents used NSAIDs but did not know of their effects on asthma and allergies, whereas 14.5% continued to use such medications despite this knowledge. As for GI side-effects, 37.4% used NSAIDs yet were aware of these particular side-effects, while 28.3% used such medications while remaining unaware of these effects. Finally, 45.7% of the respondents used NSAIDs despite understanding the risk of potential kidney disease, while 20% used them without being aware of this potential side-effect (Figure 3).

## Discussion

According to global estimates, NSAIDs comprise some of the most frequently utilized medications worldwide and taken by approximately 30 million people per day [7]. However, despite their frequency of use, NSAIDs also have the potential to cause considerable harm due to the risk of avoidable adverse drug reactions, including myocardial infarction, stroke, bleeding, and kidney damage [8,9]. NSAIDs are responsible for just under one-third of hospital admissions due to adverse drug reactions, predominantly affecting vulnerable patient populations such as the elderly, individuals taking multiple medications, and those with comorbid conditions such as cardiovascular disease, diabetes, hypertension, and pre-existing renal or liver impairment [8].

This study showed that females were most likely to use NSAIDs, as several previous studies have also reported a greater frequency of NSAID use among women [10,11], possibly because female patients are more likely to develop migraines and suffer from menstrual pain. In this study, 74.5% of the responders were younger than 50 years of age. Although this could be a limitation with underrepresentation of the older population of participants who may have higher or lower prevalence of NSAIDS use. This might not be of great importance, as previous studies have shown that younger patients are more frequent users of NSAID medications [12–14]; Also may be attributed to the fact that older people are more likely to suffer from comorbidities [15] and may therefore be wary of taking NSAIDs, the increased patient education of NSAID-related side-effects during doctor appointments could have attributed to the less use too.

Regarding tobacco use, we found smokers are less likely to use NSAIDs. It is believed that smoking might increase pain threshold and tolerance levels [16,17], thereby lowering usage of analgesics.

We also noted that 73% of our population without health insurance were NSAID users. Lack of health insurance may result in affected individuals being unable to visit physicians or having access to follow-up care, thereby leading to increased self-medication practices [18]. Moreover, respondents with comorbidities reported decreased NSAID consumption compared to those without comorbidities. This was surprising as the opposite trend was elicited in a study from Egypt, which reported that 63.5% of hypertensive patients used NSAIDs [19]. Our varying results may be explained by the fact that patients with comorbidities tend to visit physicians more often and may therefore be more educated about how such medications can worsen chronic health conditions like hypertension or diabetes [20–22].

Regarding patterns of NSAID usage, our findings showed that the most frequent indication for NSAIDs was headache (30%), similar to results reported from a study conducted in Scotland [23]. The most common frequency of usage was once per month or less often. Infrequent analgesic users should therefore be targeted for additional awareness initiatives, as well as close monitoring for possible side-effects. Our data indicated that 42.6% and 17% of users had NSAID prescriptions from a physician or pharmacist, respectively. However, 28.6% of respondents had self-prescribed themselves NSAIDs, indicating overuse of these medications in the public health system [24]. Moreover, only 29.6% of NSAID consumers in the current study were warned about the potential side-effects of such medications. A study from Switzerland found that physicians and pharmacists informed 47.3% and 25.6% of their patients, respectively, about potential side-effects [25]. Thus, healthcare professionals have an important role to play in adequately informing patients of the possible interactions and complications that may arise from NSAID utilization.

Most respondents were aware of NSAID-related kidney (65.1%) and GI (52.6%) side-effects, but few were aware of other possible side-effects such as asthma and allergies (22.4%). Similar findings have been reported among elderly citizens living in the Midwestern USA, in which 65.3% were aware of kidney problems, while only 25.0% knew of the increased risk of asthma and allergies [26]. These results indicate that awareness of NSAID-related side-effects is still inadequate in Jordan, suggesting that healthcare providers who prescribe or sell these drugs do not provide sufficient information. This could have serious implications with regards to potential side-effects, toxicity, and illness resulting in utilization of these drugs, as illustrated by a prior study showing a possible relationship between lack of awareness of proper medication use and frequency of side-effects in the Jordanian population [27]. Moreover, this is supported by a study conducted in New Zealand in which lack of knowledge about adverse effects, along with taking the maximum dosage, placed consumers at risk of NSAID-related complications [28].

According to a review of the clinical and economic burden of adverse events resulting from NSAID utilization, it is estimated that more than 100,000 patients in the USA are hospitalized every year for GI complications alone, resulting in approximately 16,500 deaths [29]. While no similar data yet exists to illustrate the burden of NSAID-related hospitalizations in the developing world, it is highly probable that complications arising from the usage of such medications cause significant morbidity and mortality. Further studies would be useful in quantifying the burden that NSAID-related complications place on the healthcare systems of developing countries.

## Conclusion

More than two-thirds of the study participants utilized NSAIDs. Most users were under 50 years of age, did not suffer from comorbidities, and were unaware of NSAID-related side-effects other than GI and renal problems. Additionally, healthcare workers must make additional efforts to educate their patients with regards to both acute problems related to NSAID utilization, such as an allergic reaction which may progress to life-threatening anaphylaxis, as well as long-term complications like kidney failure, peptic ulcer disease and elevated blood pressure with cardiovascular risk. The findings of this study may help define priorities in the Jordanian healthcare system, establish health policies on the national level, and aid in the counseling of patients regarding the appropriate use and possible adverse effects of NSAIDs.

## Data Availability

The datasets used and/or analyzed during the current study are available from the corresponding author upon reasonable request.
